# Association of functional and structural social support with chronic kidney disease among African Americans: the Jackson Heart Study

**DOI:** 10.1186/s12882-019-1432-9

**Published:** 2019-07-15

**Authors:** Rasheeda K. Hall, Clemontina A. Davenport, Mario Sims, Cathleen Colón-Emeric, Tiffany Washington, Jennifer St. Clair Russell, Jane Pendergast, Nrupen Bhavsar, Julia Scialla, Crystal C. Tyson, Wei Wang, Yuan-I Min, Bessie Young, L. Ebony Boulware, Clarissa J. Diamantidis

**Affiliations:** 1 Duke University, Durham, NC USA; 2 University of Mississippi Medical Center, Jackson, MS USA; 3 University of Georgia, Athens, GA USA; 4 University of Washington, Seattle, WA USA

**Keywords:** Minority health, Social network, Chronic renal insufficiency, Aged

## Abstract

**Background:**

There is limited evidence on the relationship between social support and renal outcomes in African Americans. We sought to determine the association of social support with prevalent chronic kidney disease (CKD) and kidney function decline in an African American cohort. We also examined whether age modifies the association between social support and kidney function decline.

**Methods:**

We identified Jackson Heart Study (JHS) participants with baseline (Exam in 2000–2004) functional and structural social support data via the Interpersonal Support Evaluation List (ISEL) and social network size questions, respectively. With ISEL as our primary exposure variable, we performed multivariable regression models to evaluate the association between social support and prevalent CKD [estimated glomerular filtration rate (eGFR) < 60 ml/min/1.73 m2 or urine albumin-creatinine ratio (ACR) ≥30 mg/g], eGFR decline, and rapid renal function decline (RRFD) (> 30% decrease in eGFR over approximately 10 years). All models were adjusted for baseline sociodemographics, diabetes, hypertension, smoking status, and body mass index; models for eGFR decline and RRFD were additionally adjusted for eGFR and ACR. In models for eGFR decline, we assessed for interaction between age and social support. For secondary analyses, we replaced ISEL with its individual domains (appraisal, belonging, self-esteem, and tangible) and social network size in separate models as exposure variables.

**Results:**

Of 5301 JHS participants, 4015 (76%) completed the ISEL at baseline. 843 (21%) had low functional social support (ISEL score < 32). Participants with low (vs. higher) functional social support were more likely to have lower income (47% vs. 28%), be current or former tobacco users (39% vs. 30%), have diabetes (25% vs. 21%) or CKD (14% vs. 12%). After multivariable adjustment, neither ISEL or social network size were independently associated with prevalent CKD, eGFR decline, or RRFD. Of the ISEL domains, only higher self-esteem was associated with lower odds of prevalent CKD [OR 0.94 (95% CI 0.89–0.99)]. The associations between social support measures and eGFR decline were not modified by age.

**Conclusions:**

In this African-American cohort, social support was not associated with prevalent CKD or kidney function decline. Further inquiry of self-esteem’s role in CKD self-management and renal outcomes is warranted.

**Electronic supplementary material:**

The online version of this article (10.1186/s12882-019-1432-9) contains supplementary material, which is available to authorized users.

## Background

African Americans bear a disproportionate burden of chronic kidney disease (CKD) [1]. Factors that contribute to this health disparity include sociodemographics, quality or access to care, and lifestyle, while nearly half of this excess risk is unknown [2]. One related psychosocial factor that may influence this excess risk is social support [3]. Social support refers to perceived emotional, material, or informational resources provided by others and/or size of one’s social network [4]. The presence of adequate social support is associated with lower risk of morbidity and mortality in the general population [5–11]. This lower risk is explained by social support’s facilitation of health-promoting behaviors [12]. In African Americans, social support buffers against the long-term health effects of stress, including stressful experiences of perceived discrimination [13], in addition to its association with improved glycemic control and blood pressure in this population [14, 15]. Thus evidence suggests that social support may have a meaningful association with CKD outcomes in African Americans.

Social support influences chronic disease self-management via both functional and structural pathways [11]. Functional social support is based on the stress-buffering hypothesis, and refers to specific resources provided by a person’s social network (e.g., emotional support, socialization, financial assistance, advice) that can help them cope with life stress and gain confidence in managing their chronic disease, which positively impacts physiological processes [12, 16]. Structural social support, based on social network theory that isolation negatively impacts health, refers to the size of a person’s social network (i.e., number of people with whom one has meaningful and frequent contact) available to provide functional social support [5]. Prior studies in African Americans show both functional and structural social support are associated with both improved health outcomes and self-management behaviors [14, 15, 17, 18]. In a cohort of African Americans with hypertensive CKD, social support was significantly higher among those who reported better quality of life [19]. However, it remains unknown whether functional or structural social support are individually associated with CKD outcomes.

The objective of this study is to determine the association of both functional and structural social support with prevalent CKD and kidney function decline using data from the Jackson Heart Study (JHS), an all African American cohort study. Our hypothesis is that low social support would be associated with prevalent CKD and greater kidney function decline. Because there is a larger burden of disability, cognitive impairment, and social isolation among older adults [20], older adults may be more vulnerable to the effects of social support on self-management behaviors that mitigate risk of kidney function decline [21, 22]. Therefore, we also examined the extent to which age modifies the association of social support with kidney function decline.

## Methods

### Study design and population

We conducted an observational study of social support and kidney function outcomes in African Americans using data from JHS participants. The JHS is a community-based cohort study designed to evaluate heart disease risk factors in African Americans residing in the tri-county area (Hinds, Rankin, and Madison) of Jackson, Mississippi [23–25]. Briefly, 5301 African Americans ages 21 to 94 years old enrolled and participated in an initial study evaluation between 2000 and 2004 (Exam 1) and subsequent follow-up evaluations between 2005 and 2008 (Exam 2) and 2009–2013 (Exam 3). The JHS protocol was approved by the Institutional Review Boards at Mississippi Medical Center, Jackson State University, and Tougaloo College, and all participants provided informed consent.

For these analyses, we included JHS participants who completed social support measures and laboratory tests of kidney function at Exam 1. Those missing these data were excluded. For analyses of kidney function decline, we limited the cohort to JHS participants with diabetes, hypertension and/or CKD at Exam 1 and excluded JHS participants who reported a history of dialysis or kidney transplant at any visit (to avoid uncertainty about the accuracy of reported eGFR values).

### Primary independent variable: social support

Our primary exposure of interest was functional social support, the nature by which interpersonal relationships provide support, assessed using the Interpersonal Support Evaluation List (ISEL) instrument. The ISEL is a validated self-administered instrument comprised of Likert-type items that represent four domains of functional social support, which include: 1) appraisal, 2) belonging, 3) self-esteem, and 4) tangible [26, 27]. The appraisal domain measures perceived availability of a confidant to talk to about one’s problems. The belonging domain measures perceived availability of people with whom one can do things. The self-esteem domain measures the extent to which someone views themselves positively when comparing one’s self with others. The tangible domain measures perceived availability of material aid, such as financial or transportation assistance. Each of the 4 domains contributes 12 points. Using the 16-item ISEL instrument, we used a standardized approach to compute summed ISEL scores (range 0 to 48) [27]. When used as a dichotomous variable, an ISEL score < 32 was used to indicate low functional social support [8].

We secondarily assessed structural social support, often assessed by social network size beyond immediate family members. Developed for this study, we assessed social network size from three items adapted from the previously validated Berkman Social Network Index (Cronbach’s α from our analytic dataset was 0.73) [5, 6]: 1) “how many close friends do you have?”, 2) “how many close relatives do you have?”, and 3) “how many of these friends or relatives do you see at least once a month?”. Possible responses for each question were: “none”, “1 or 2”, “3 to 5”, “6 to 9”, or “10 or more”. We designated a score to each item ranging from 0 to 4, respectively. We then computed an overall social network size as a sum of all three items, ranging from 0 to 12 to quantify structural social support. Aligning with a two-thirds cutoff to indicate low social support using the ISEL, social network size < 8 was used to categorize participants as having low structural social support. Both the ISEL and social network size questions were provided to JHS participants during the initial home interview.

### Covariates

We identified the following potential confounders of the relationship between social support and kidney function outcomes: 1) demographics (age, sex), 2) socioeconomic status (education [defined as ≤ high school degree vs. > high school degree], income [defined as income ≤1.5 times the poverty level vs. > 1.5 times the poverty level and an additional category for missing income level]), 3) diabetes (defined as fasting glucose ≥126 mg/dL or HbA1c ≥6.5% or actual use of diabetic medications), 4) hypertension (defined as blood pressure [BP] > 140/90 or self-report use of BP lowering meds), 5) smoking status (active/former vs. never-smoker based on self-report), and 6) body mass index (BMI) defined as kg/m^2^.

### Primary dependent variable: kidney function

The outcomes were 1) prevalent CKD and 2) estimated glomerular filtration rate (eGFR) decline over follow-up. We defined prevalent CKD as an eGFR < 60 ml/min/1.73m^2^ using the Chronic Kidney Disease-Epidemiology Collaboration (CKD-EPI) estimating equation^11^ or a urine albumin-to-creatinine ratio (ACR) ≥30 mg/g at Exam 1. We defined decline in eGFR as the annualized rate of change in eGFR (ml/min/1.73m^2^) from Exam 1 to Exam 3; based on the following equation: 365.25 days × (eGFR_Exam1_ – eGFR_Exam3_)/(number of days between Exam 1 and Exam 3).

### Statistical analysis

We used two-sample t-tests for continuous variables or chi-squared tests for categorical variables to compare sociodemographic and clinical characteristics of study participants in our analytic cohort to those excluded for missing ISEL data. We used the same statistical tests to compare sociodemographic and clinical characteristics of study participants with low vs. high functional social support as defined by our primary exposure variable, ISEL. Because these tests were not part of our primary aims and hypotheses, we did not adjust for multiple testing. To evaluate the association of social support based on both ISEL or social network size with our outcomes of interest, we evaluated each social support measure in separate models as either continuous (ISEL score or social network size) or categorical variables (low vs. high social support). Multivariable logistic regression models used to determine the relationship between social support and prevalent CKD were adjusted for age, sex, income, education, BMI, smoking status, diabetes, and hypertension. Multivariable linear regression models to determine the relationship between social support and eGFR decline were additionally adjusted for baseline ACR and eGFR. We added an interaction term [ISEL (or social network size) × age] to examine if age moderated the effect of social support on eGFR decline.

In secondary analyses, we stratified our models by marital status as marital status can have a significant role in social support but is not measured in either ISEL or social network size [5]. Recognizing the measurement bias of determining annual change in kidney function decline with only two timepoints, we added a secondary outcome, rapid renal function decline (RRFD). We defined RRFD as a > 30% decline in eGFR between Exams 1 and 3, an approximately ten-year period. Multivariable logistic regression models used to determine the relationship between social support and RRFD had the same adjustment variables as the models for eGFR decline. To identify associations with specific aspects of social support, we also evaluated each social support domain (for social network size, individual questions) as the exposure variables. All analyses were performed with SAS Version 9.4 (SAS Institute, Cary NC) and R 3.4.0 (R Core Team, Vienna Austria) and used a two-sided 0.05 level for statistical significance.

## Results

### Cohort characteristics

**Fig. 1:**
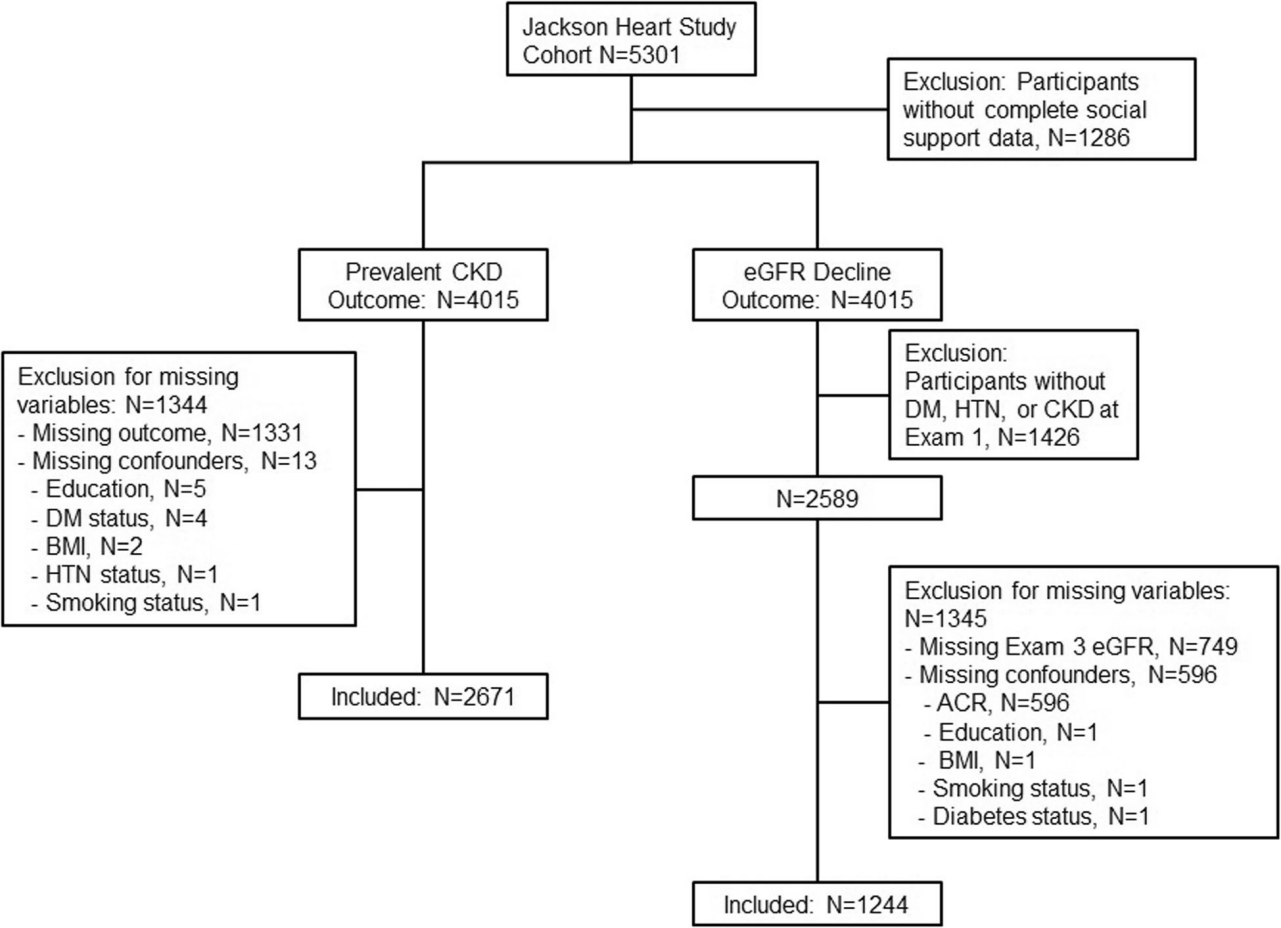
Flow Diagram of Selection of Analytic Cohorts. The analytic cohort for eGFR decline differs from the analytic cohort for prevalent CKD because of exclusion of participants without known diabetes, hypertension or CKD at baseline

Of 5301 JHS participants, 4015 (76%) completed the ISEL at baseline. We excluded 1344 participants because of missing kidney function measures or covariates at baseline, leaving 2671 in our analytic cohort for the prevalent CKD outcome model. Similarly, we identified 2589 participants with diagnoses of diabetes, hypertension or CKD at baseline, but excluded 1345 participants because of missing kidney function measures and/or covariates, leaving 1244 in our analytic cohort for the kidney function decline outcome models (eGFR decline and RRFD) (Fig. 1). Compared to JHS participants who did not complete ISEL at baseline, those who did complete ISEL were more likely to have high school education (64% vs. 52%) and income above poverty level (53% vs. 43%), and less likely to have hypertension (59% vs. 63%), diabetes (21% vs. 24%), and CKD (12% vs. 15%). (Table 1). Characteristics of participants with and without CKD are shown in Additional file 1: Table S1.

**Table 1:** Comparison JHS Participants Included and Excluded in Analyses

	Overall (*n* = 5301)	Included (*n* = 4015)	Excluded (*n* = 1286)	*P*-value
*Demographics*				
Age, year	55.4 ± 12.8	54.8 ± 12.6	57.2 ± 13.6	< 0.01
Men	1934 (36.5)	1436 (35.8)	498 (38.7)	0.06
High school education	3232 (61)	2567 (63.9)	665 (51.7)	< 0.01
Income				< 0.01
≤ 1.5 times the poverty level	1798 (33.9)	1297 (32.3)	501 (39)	
> 1.5 times the poverty level	2683 (50.6)	2130 (53.1)	553 (43)	
Missing	820 (15.5)	588 (14.6)	232 (18)	
*Renal Disease Risk Factors*				
Body Mass Index	31.8 ± 7.2	31.8 ± 7.2	31.6 ± 7.3	0.52
Smoking status				0.07
Never	3574 (67.4)	2738 (68.2)	836 (65)	
Former or current	1716 (32.4)	1275 (31.8)	441 (34.3)	
Hypertension (*n* = 5278)	3169 (60.0)	2365 (58.9)	804 (62.5)	< 0.01
Diabetes (*n* = 5240)	1152 (21.9)	845 (21.0)	307 (23.9)	0.01
*Renal Function*				
Annual eGFR decline	1.3 ± 2	1.2 ± 2	1.4 ± 2	0.09
eGFR at Visit 1	94.2 ± 22	94.9 ± 21.5	91.7 ± 23.3	< 0.01
logACR at Visit 1	2.2 ± 1.3	2.1 ± 1.2	2.3 ± 1.3	< 0.01
Albuminuria at Visit 1				0.16
No	2875 (54.2)	2287 (57)	588 (45.7)	
Yes	429 (8.1)	328 (8.2)	101 (7.9)	
Missing	1997 (37.7)	1400 (34.9)	597 (46.4)	
CKD at Visit 1				< 0.01
No	2749 (51.9)	2197 (54.7)	552 (42.9)	
Yes	673 (12.7)	487 (12.1)	186 (14.5)	
Missing	1879 (35.4)	1331 (33.2)	548 (42.6)	
*Social Support*				
Functional (ISEL score)	37 ± 7.2	37 ± 7.2	35.8 ± 7.1	0.30
Structural (Social network size)	5.9 ± 2.7	5.9 ± 2.7	6 ± 2.7	0.28

Data expressed as *n* (%) or mean ± SD

### Social support

The average ISEL score was relatively high, 37.0 (SD = 7.2), and 21.0% (*n* = 843) of participants reported low functional social support (ISEL < 32). Among those with low functional social support, 85.3% (*n* = 719) also had low structural social support (social network size < 8). Participants with low functional social support (vs. higher) were more likely to have lower income (47% vs. 28%), be current or former tobacco users (39% vs. 30%), and to have diabetes (25% vs. 21%) (Table 2).

**Table 2:** Baseline Characteristics of analytic sample by level of social support

Variable	Total (*N* = 4015)	Low Social Support ISEL < 32 (*N* = 843)	High Social Support ISEL ≥32 (*N* = 3172)	*P*-value
*Demographics*				
Age	54.8 ± 12.6	54.8 ± 13.2	54.8 ± 12.4	0.87
Men	1436 (35.8)	307 (36.4)	1129 (35.6)	0.69
High school education	2567 (63.9)	430 (51)	2137 (67.4)	< 0.01
Income				< 0.01
≤ 1.5 times the poverty level	1297 (32.3)	400 (47.4)	897 (28.3)	
> 1.5 times the poverty level	2130 (53.1)	319 (37.8)	1811 (57.1)	
Missing	588 (14.6)	124 (14.7)	464 (14.6)	
*Renal Disease Risk Factors*				
Body Mass Index (*n* = 4008)	31.8 ± 7.2	32.5 ± 7.6	31.6 ± 7.1	< 0.01
Smoking status				< 0.01
Never	2738 (68.2)	518 (61.4)	2220 (70)	
Former or current	1275 (31.8)	325 (38.6)	950 (29.9)	
Missing	2 (0)	0 (0)	2 (0.1)	
Hypertension				0.11
No HTN	1631 (40.6)	322 (38.2)	1309 (41.3)	
Controlled HTN	1198 (29.8)	254 (30.1)	944 (29.8)	
Uncontrolled HTN	1167 (29.1)	262 (31.1)	905 (28.5)	
Have HTN but can’t determine control^a^	16 (0.4)	5 (0.6)	11 (0.3)	
Missing	3 (0.1)	0 (0)	3 (0.1)	
Diabetes				0.03
No DM	3129 (77.9)	633 (75.1)	2496 (78.7)	
Controlled DM	422 (10.5)	107 (12.7)	315 (9.9)	
Uncontrolled DM	423 (10.5)	98 (11.6)	325 (10.2)	
Missing	41 (1)	5 (0.6)	36 (1.1)	
*Renal Function*				
Annual renal function decline (*n* = 2947)	1.2 ± 2	1.3 ± 2	1.2 ± 2	0.8
eGFR at Visit 1 (*n* = 3953)	94.9 ± 21.5	94.5 ± 24.2	95.1 ± 20.7	0.51
logACR at Visit 1 (*n* = 2614)	2.1 ± 1.2	2.2 ± 1.3	2.1 ± 1.2	0.30
Albuminuria at Visit 1				0.15
No	2287 (57)	442 (52.4)	1845 (58.2)	
Yes	328 (8.2)	75 (8.9)	253 (8)	
Missing	1400 (34.9)	326 (38.7)	1074 (33.9)	
CKD at Visit 1				0.02
No	2197 (54.7)	423 (50.2)	1774 (55.9)	
Yes	487 (12.1)	118 (14)	369 (11.6)	
Missing	1331 (33.2)	302 (35.8)	1029 (32.4)	
*Social Support*				
Functional (ISEL score)	37 ± 7.2	26.1 ± 4.7	39.9 ± 4.4	–
Structural (Social network size)	5.88 ± 2.67	4.81 ± 2.45	6.17 ± 2.65	< 0.01

Data expressed as *n* (%) or mean ± SD based on total of 4015 participants, unless otherwise specified

^a^Unable to identify if achieve Hypertension control in participants with missing diabetes status and BP between 130/80 and 140/90

### Functional social support and kidney function

Prevalent CKD was more common among participants with low (vs. high) functional social support (22% vs 17%). However, among the 2589 participants with diabetes, hypertension or CKD at baseline, there was no difference in annual rate of eGFR decline between participants with low vs. high functional social support (1.3 ± 2.2 ml/min/1.73m^2^ vs. 1.4 ± 2.1 ml/min/1.73m^2^). In multivariable regression modeling, we found no association between functional social support (total ISEL score) and prevalent CKD (OR 0.99, 95% CI 0.98,1.01) or eGFR decline (β = − 0.01, 95% CI -0.02, 0.01) (Table 3). These findings were unchanged in models with dichotomized ISEL as the exposure variable: prevalent CKD (OR 1.14, 95% CI 0.88, 1.48) and eGFR decline (β = 0, 95% CI -0.28,0.28). Age did not modify the relationship between functional social support and eGFR decline (*p* = 0.71). Of the models with each ISEL subscale as exposure variables; we found the ISEL self-esteem domain score was associated with prevalent CKD (OR 0.94, 95% CI 0.89,0.99), and the ISEL appraisal and tangible domain scores had significant but modest associations with eGFR decline (β = − 0.07, 95% CI − 0.13, 0) and (β = 0.07, 95% CI 0.01, 0.13), respectively (Table 3). Total ISEL score was not associated with prevalent CKD (OR 1.00, 95% CI 0.98, 1.02) or eGFR decline (B = 0, 95% CI -0.02, 0.02) in models stratified by marital status or with RRFD (OR 1.00, 95% CI 0.98,1.02).

**Table 3:** Adjusted Analyses of Social Support and Kidney Function

Variable	Relative Odds of Prevalent CKD^a^ OR (95% CI)	Difference in eGFR decline^b^ β (95% CI)
Functional Social Support		
ISEL Total^c^	0.99 (0.98, 1.01)	−0.01 (−0.02, 0.01)
ISEL Subscales		
Appraisal	1.03 (0.96, 1.10)	**−0.07 (−0.13, 0.00)**
Belonging	0.99 (0.93, 1.05)	−0.02 (− 0.09, 0.04)
Self-esteem	**0.94 (0.89, 0.99)**	−0.02 (− 0.08, 0.04)
Tangible	1.01 (0.95, 1.07)	**0.07 (0.01, 0.13)**
Structural Social Support		
Social network size score^d^	0.99 (0.95, 1.03)	0 (−0.04, 0.04)

Table reflects effect estimates of separate multivariable regression models using ISEL total score, ISEL subscales, and social network size score as exposure variables of interest. Data expressed as effect estimate [odds ratio (OR) or beta coefficient] and 95% confidence interval (CI). Significant effect estimates (*p* < .05) indicated in bold

^a^Model 1 has prevalent CKD as the outcome. Analyses conducted on complete cases, *n* = 2671, and were adjusted for age, gender, income, education, body mass index, smoking status, diabetes, and hypertension

^b^Model 2 has eGFR decline in ml/min/1.73m^2^ per year as the outcome. Analyses conducted on complete cases, *n* = 1244 (participants with diabetes, hypertension or CKD at baseline), and were adjusted for age, gender, income, education, body mass index, smoking status, diabetes, and hypertension, albumin/creatinine ratio, and baseline eGFR

^c^Interpersonal Support Evaluation List (ISEL) is a validated measure of functional social support

^d^Social network size is a measure of structural social support based on number and frequency of close contacts

### Structural social support and kidney function

In analyses of our secondary exposure variable, social network size, there was no association with prevalent CKD (OR 0.99, 95% CI 0.95,1.03) or eGFR decline (β = 0.00, 95% CI -0.04, 0.04). (Table 3). These findings were unchanged in models with dichotomized social network size as the exposure variable: prevalent CKD (OR 1.06, 95% CI 0.84, 1.34) and eGFR decline (β = − 0.02, 95% CI -0.25,0. 22). Age did not modify the relationship between structural social support and eGFR decline (*p* = 0.72). Of models with individual social network items as the exposure variables, we found no association between individual social network items and prevalent CKD or eGFR decline. Social network size was not associated with prevalent CKD (OR 1.00, 95% CI 0.94, 1.06) or eGFR decline (β = 0.01, 95% CI -0.05, 0.07) in models stratified by marital status or with RRFD (OR 1.00, 95% CI 0.95,1.06).

## Discussion

In this cohort of African American adults, we found that participants with low functional and/or structural social support were not more likely to have CKD. Additionally, among participants with CKD or at high risk due to diabetes and hypertension, high levels of functional and structural social support were not associated with faster decline in kidney function. However, within individual domains, higher ISEL self-esteem domain score appeared to be associated with lower odds of prevalent CKD. Although modest, this finding suggests that further study into the role of self-esteem in CKD outcomes could uncover novel interventions targeting CKD among African Americans.

To our knowledge, this is the first study to examine the extent to which social support is independently associated with CKD outcomes in an African American cohort. Two observational studies of patients receiving dialysis demonstrate high social support was independently associated with improved survival [7, 10]. One cross-sectional study in African Americans identified high social support to be independently associated with modest reduction in odds of diabetes and hypertension [18]. A separate study conducted in African Americans demonstrated that increasing social support was associated with more self-management behavior (e.g., adding more fruits and vegetables to meal plan or counting number of fruits or vegetables eaten per day) and subsequently more fruit and vegetable consumption [17]. In contrast to those studies, we did not identify an independent relationship between functional and structural measures of social support and CKD outcomes. However, our study adds to these findings by demonstrating that high self-esteem, as it relates to social support, has a modest independent association with lower odds of prevalent CKD. The associations between the tangible and appraisal domains of functional social support and kidney function decline were too modest to represent a clinically meaningful change in eGFR.

Our findings may be explained, in part, by our study design. Although functional and structural social support have been associated with some clinical outcomes, it may have been more appropriate to measure the association between CKD and specific self-management behaviors. Self-management behaviors, such as physical activity or adherence to diabetes and hypertension medications, could be in the causal pathway between social support and CKD outcomes [28]. Thus, additional steps to identify an association between social support and self-management behaviors is warranted. Our findings may also be influenced by the low prevalence of CKD (13%) and low social support (21%) in the JHS. Most importantly, our findings may be explained by the appropriateness of our exposure variables, the ISEL and social network measures, in this African American cohort. The ISEL used in our study has been administered in other studies with African American participants, with similar mean total score (ranging from 30 to 37) [19, 27]. However, the survey’s items may be subject to measurement bias. Most participants reported high scores on ISEL items which may represent social desirability bias, or response bias that occurs when respondents want to give responses that are socially acceptable [29]. Alternatively, the ISEL items may not account for the cultural nuances that tend to shape how African Americans may perceive their level of social support [9]. Our assessment of social network size was limited in its ability to discretely assess additional aspects of structural social support (e.g., church membership, social group involvement) that are captured in the Berkman social network index [5, 6]. Overall, these findings, along with evidence from other studies examining psychosocial factors in African Americans, endorse additional research on the content validity of psychosocial instruments in this population.

We did find that higher scores on the ISEL self-esteem domain were associated with a modest reduction in odds of prevalent CKD. The ISEL self-esteem domain assesses an individual’s ability to have a positive comparison when comparing oneself with others, and it is correlated with the widely used Rosenberg self-esteem scale [30]. It is plausible that individuals who have higher perceived self-esteem social support are less likely to have CKD in settings where this high self-esteem is tied with high self-efficacy. Individuals with high self-efficacy are more likely to engage in health behaviors and lifestyle modifications that can prevent onset and/or progression of CKD [31]. If self-esteem from social support is truly a buffer against development of CKD, we would expect that the self-esteem score would also be associated with a slower decline in eGFR. Absence of that finding in this study does not rule out the possibility that some individuals with risk factors for CKD, like diabetes or hypertension, may possess some level of psychosocial resilience that buffers against the onset of CKD. A longitudinal cohort study that evaluates changes in both social support and health status over time may uncover a temporal relationship between self-esteem social support and kidney function decline. More evidence supporting this relationship could increase the argument for behavioral interventions that aim to enhance self-esteem social support in African Americans with risk factors for CKD.

The important strengths of this study are its: 1) large African American community-based cohort, 2) rich psychosocial assessment data to assess both functional and structural social support in CKD, and 3) longitudinal measures of kidney function. Despite these strengths, our study has its limitations. First, the cross-sectional study design limits our ability to make any causal or temporal inference about the association of social support, specifically self-esteem, with prevalent CKD. For models of eGFR decline, we restricted the analytic cohort to participants with baseline CKD, diabetes, or hypertension. While those comorbidities are important risk factors for eGFR decline, these comorbidities may have concealed an association of social support with eGFR decline. Also, we used social support measures that have not been formally validated to confirm the best cutoff for low social support. Despite this limitation, these variables as continuous measures did not demonstrate an association with kidney function outcomes. We were unable to account for unmeasured confounders, such as changes in health status and social support over time. It is plausible that lack of association in those models may be explained, in part, by an increase in perceived social support in the time between research visits and/or limited sample size. Last, we examined data from research volunteers so these findings have limited generalizability to all African Americans.

## Conclusion

In an African American community-based cohort study, functional or structural social support measures were not independently associated with CKD outcomes. However, self-esteem measures showed a modest independent association with prevalent CKD. Because of strong evidence underlying the role of psychosocial factors in chronic disease self-management, there remains a need for additional research on the type and extent of social support that may be modified to improve kidney function outcomes in African Americans.

## Additional file

Additional file 1: **Table S1.** Comparison of characteristics of JHS participants with and without CKD. (DOCX 19 kb)

## Data Availability

The data that support the findings of this study are available from The Jackson Heart Study but restrictions apply to the availability of these data, which were used under license for the current study, and so are not publicly available. Data are however available from the authors upon reasonable request and with permission of The Jackson Heart Study.

## Abbreviations

ACR: Albumin-to-creatinine ration
CI: Confidence interval
CKD: Chronic kidney disease
EGFR: Estimated glomerular filtration rate
ISEL: Interpersonal Support Evaluation List
JHS: Jackson Heart Study
OR: Odds ratio
RRFD: Rapid renal function decline
