# Supplementary material for: Association of functional and structural social support with chronic kidney disease among African Americans: the Jackson Heart Study
Source: BMC Nephrol. 2019 Jul 15;20:262. doi: 10.1186/s12882-019-1432-9 (PMC6633656; doi:10.1186/s12882-019-1432-9)
Supplement: Supplementary file 1 — Table S1. Comparison of characteristics of JHS participants with and without CKD. (DOCX 19 kb) [file 12882_2019_1432_MOESM1_ESM.docx]

Additional file 1: Table S1. Characteristics of JHS Participants based on CKD status

|  | Total | No CKD | CKD | Unknown^a^ | P |
| --- | --- | --- | --- | --- | --- |
| Variable | (N=4015) | (N=2197) | (N=487) | (N=1331) |  |
| *Demographics* |  |  |  |  |  |
| Age | 54.8 ± 12.6 | 52.6 ± 12.3 | 61.1 ± 12.7 | 56 ± 11.9 | 0 |
| Men | 1436 (35.8) | 805 (36.6) | 168 (34.5) | 463 (34.8) | 0.4 |
| High school education | 2567 (63.9) | 1533 (69.8) | 246 (50.5) | 788 (59.2) | 0 |
| Income |  |  |  |  | 0 |
| ≤ 1.5 times the poverty level | 1297 (32.3) | 608 (27.7) | 197 (40.5) | 492 (37) |  |
| > 1.5 times the poverty level | 2130 (53.1) | 1233 (56.1) | 206 (42.3) | 691 (51.9) |  |
| Missing | 588 (14.6) | 356 (16.2) | 84 (17.2) | 148 (11.1) |  |
| *Renal Disease Risk Factors* |  |  |  |  |  |
| Body Mass Index (*n*=4008) | 31.8 ± 7.2 | 31.5 ± 7 | 33.3 ± 7.5 | 31.7 ± 7.4 | 0 |
| Smoking status |  |  |  |  | 0 |
| Never | 2738 (68.2) | 1575 (71.7) | 324 (66.5) | 839 (63) |  |
| Former or current | 1275 (31.8) | 621 (28.3) | 163 (33.5) | 491 (36.9) |  |
| Missing | 2 (0) | 1 (0) | 0 (0) | 1 (0.1) |  |
| Hypertension |  |  |  |  | 0 |
| No HTN | 1631 (40.6) | 1009 (45.9) | 76 (15.6) | 546 (41) |  |
| Controlled HTN | 1198 (29.8) | 650 (29.6) | 176 (36.1) | 372 (27.9) |  |
| Uncontrolled HTN | 1167 (29.1) | 536 (24.4) | 233 (47.8) | 398 (29.9) |  |
| Have HTN but can’t determine control^b^ | 16 (0.4) | 1 (0.05) | 1 (0.2) | 14 (1.0) |  |
| Missing | 3 (0.1) | 1 (0.05) | 1 (0.2) | 1 (0.1) |  |
| Diabetes |  |  |  |  | 0 |
| No DM | 3129 (77.9) | 1843 (83.9) | 272 (55.9) | 1014 (76.2) |  |
| Controlled DM | 422 (10.5) | 207 (9.4) | 89 (18.3) | 126 (9.5) |  |
| Uncontrolled DM | 423 (10.5) | 147 (6.7) | 122 (25.1) | 154 (11.6) |  |
| Missing | 41 (1) | 0 (0) | 4 (0.8) | 37 (2.8) |  |
| *Renal Function* |  |  |  |  |  |
| Annual renal function decline (*n*=2947) | 1.2 ± 2 | 1.1 ± 1.8 | 1.9 ± 2.6 | 1.3 ± 2 | 0 |
| eGFR at Visit 1 (*n*=3953) | 94.9 ± 21.5 | 99.2 ± 17.6 | 73.3 ± 31.8 | 95.7 ± 17.7 | -- |
| logACR at Visit 1 (*n*=2614) | 2.1 ± 1.2 | 1.7 ± 0.6 | 4.3 ± 1.5 | 1.9 ± 0.6 | -- |
| Albuminuria at Visit 1 |  |  |  |  | -- |
| No | 2287 (57) | 2197 (100) | 63 (12.9) | 27 (2) |  |
| Yes | 328 (8.2) | 0 (0) | 328 (67.4) | 0 (0) |  |
| Missing | 1400 (34.9) | 0 (0) | 96 (19.7) | 1304 (98) |  |
| CKD at Visit 1 |  |  |  |  |  |
| No | 2197 (54.7) | 2197 (100) | 0 (0) | 0 (0) | -- |
| Yes | 487 (12.1) | 0 (0) | 487 (100) | 0 (0) |  |
| Missing | 1331 (33.2) | 0 (0) | 0 (0) | 1331 (100) |  |
| *Social Support* |  |  |  |  |  |
| Structural (Social network size) | 5.9 ± 2.7 | 5.9 ± 2.6 | 6.1 ± 2.8 | 5.8 ± 2.7 | 0.1 |
| Functional (ISEL score) | 37 ± 7.2 | 37.4 ± 7.1 | 36.5 ± 7.1 | 36.6 ± 7.3 | 0 |

Data expressed as n (%) or mean ± SD based on total of 4,015 participants, unless otherwise specified.

^a^Unknown indicates JHS participants without laboratory data for determining CKD status.

^b^Unable to identify if achieve Hypertension control in participants with missing diabetes status and BP between 130/80 and 140/90.
